# A Comparative Neuro-Histological Assessment of Gluteal Skin Thickness and Cutaneous Nociceptor Distribution in Horses and Humans

**DOI:** 10.3390/ani10112094

**Published:** 2020-11-11

**Authors:** Lydia Tong, Melinda Stewart, Ian Johnson, Richard Appleyard, Bethany Wilson, Olivia James, Craig Johnson, Paul McGreevy

**Affiliations:** 1 Taronga Conservation Society Australia, Mosman, Sydney, NSW 2088, Australia; ltong@zoo.nsw.gov.au; 2 Starling Scientific, Pearl Beach, NSW 2256, Australia; starling.scientific18@gmail.com; 3 Faculty of Medicine, Health and Human Sciences, Macquarie University, Sidney, NSW 2109, Australia; ian.johnson@mq.edu.au (I.J.); richard.appleyard@mq.edu.au (R.A.); 4 Sydney School of Veterinary Science, University of Sydney, Sydney, NSW 2006, Australia; bethany.wilson@sydney.edu.au; 5 Australian Veterinary Equine Dentistry, 27 Bellevue Terrace, Clayfield, QLD 4011, Australia; dr.olivia.james@gmail.com; 6 School of Veterinary Science, Tāwharau Ora, Massey University, Private Bag 11 222, Palmerston North 4442, New Zealand; c.b.johnson@massey.ac.nz

**Keywords:** innervation, dermis, epidermis, nerve cell counts, pain, whipping

## Abstract

**Simple Summary:**

This study was performed to increase the understanding of the capacity of horse skin to detect pain when directly compared to human skin. The study focused on gluteal skin where horses are most often struck with whips during racing. The study was designed to inform the debate surrounding the use of whip strikes in horse racing where there is increasing pressure on the global racing industry to justify whip use. At the core of the debate is the question—do horses experience pain when being whipped? The study used microscopic studies of skin from 10 deceased humans and 20 euthanased horses to explore any differences between the species in their skin structure and nerve supply. The results revealed no significant difference between humans and horses in either the concentration of nerve endings in the outer pain-detecting layer of skin (epidermis) or in the thickness of this layer. In horses, this layer was deeper on the right than on the left. The collagen layer (dermis) of skin which is not involved in pain detection was significantly thinner in humans than in horses. These findings show that, although horse skin is thicker overall than human skin, the part of the skin that is thicker does not insulate them from pain that is generated during a whip strike, and that humans and horses have the equivalent basic anatomic structures to detect pain in the skin.

**Abstract:**

The current project aims to build on knowledge of the nociceptive capability of equine skin to detect superficial acute pain, particularly in comparison to human skin. Post-mortem samples of gluteal skin were taken from men (*n* = 5) and women (*n* = 5), thoroughbreds and thoroughbred types (mares, *n* = 11; geldings, *n* = 9). Only sections that contained epidermis and dermis through to the hypodermis were analysed. Epidermal depth, dermal depth and epidermal nerve counts were conducted by a veterinary pathologist. The results revealed no significant difference between the epidermal nerve counts of humans and horses (*t* = 0.051, *p* = 0.960). There were no significant differences between epidermal thickness of humans (26.8 µm) and horses (31.6 µm) for reference (left side) samples (*t* = 0.117, *p* = 0.908). The human dermis was significantly thinner than the horse dermis (*t* = −2.946, *p* = 0.007). Epidermal samples were thicker on the right than on the left, but only significantly so for horses (*t* = 2.291, *p* = 0.023), not for humans (*t* = 0.694, *p* = 0.489). The thicker collagenous dermis of horse skin may afford some resilience versus external mechanical trauma, though as this is below the pain-detecting nerve endings, it is not considered protective from external cutaneous pain. The superficial pain-sensitive epidermal layer of horse skin is as richly innervated and is of equivalent thickness as human skin, demonstrating that humans and horses have the equivalent basic anatomic structures to detect cutaneous pain. This finding challenges assumptions about the physical capacity of horses to feel pain particularly in comparison to humans, and presents physical evidence to inform the discussion and debate regarding the ethics of whipping horses.

## 1. Introduction

The use of whips in horse racing is increasingly being questioned on ethical, welfare [1,2,3,4,5,6,7,8,9,10,11], social sustainability [10], and legal grounds [8]. Racing industry proponents argue that “the whip is used for safety (of both rider and horse) or to encourage the horse to perform to its best when in contention”, and that horses in many jurisdictions are protected from pain by whip padding and rules that govern whip use [12]. Some industry commentators have asserted that racing horses are immune to any pain from whip strikes chiefly because they are adrenalinised [13]. Others point out that horses may be physically brutal with one another in social interactions or conflict [14] and have therefore evolved to be robust and stoic. At the core of the debate is a fundamental question—do horses experience pain when whipped?

It is broadly accepted in science and society that most animals experience pain when struck. This is reflected by current societal norms, ethics, and laws that prohibit the striking or hitting animals. However, this protection is not extended to certain animal species—these being almost exclusively large domestic species—including the horse. It is reasonable to question why the assumption exists, and crucially, what scientific evidence exists to demonstrate that horses do or do not feel pain when being whipped.

Assumptions that horses are insensitive to pain may be influenced by historic factors and colloquialisms. The term “thick-skinned”, to denote insensitivity, has been in the English lexicon for over 400 years [15]. In parallel, the scientific community previously taxonomically defined and grouped certain species by having thick skin. The term “pachyderm” (from the Greek “pachys”, meaning “thick” and “derma”, meaning “skin”) long grouped elephants, hippopotamus, horses, pigs, and other large species together [16].

In recent decades, limited scientific data have become available that document the full skin thickness of horses, with it reportedly ranging between 1.2 to 7 mm. [17,18]. This is indeed considerably thicker than full thickness human skin, reported at 1.2 mm thick on average in multiple studies [19,20,21], with a range of 0.52 to 2.0 mm [21]. However, interpreting the skin thickness as a whole as an indicator of skin sensitivity fails to account for the complexity of the skin organ and, crucially, that detection of pain by mammalian skin overwhelmingly occurs in the very superficial layer of the skin (the epidermis), where the source of the painful stimulus (e.g., a strike or a heat source) comes into contact with these cells. Pain receptors of the skin are, in the vast majority, found in the epidermis, terminating in the mid to superficial epidermis [22,23]. These are fine, non-myelinated fibrils that detect damage to epidermal cells [22]. There may also be some dermal structures that have potential to act as nociceptors under some circumstances, such as thermal noxious stimulation [24] and following experimental selective ablation of epidermal nociceptors [25].

Reported data on horse epidermal thickness are sparse, though a mean epidermal thickness of 53 μm has been reported [26]. A recent study reported depth of equine epidermal from the thorax as 29 μm and from the limbs as 46 μm [27]. In comparison, four studies of human epidermal thickness found a mean epidermal depth of 51.2, 52.2, 53.0, and 91.8 μm, respectively [19,20,21,28]. What these data suggested is that where it counts, in terms of pain sensitivity, horse skin may be no more meaningfully thick than human skin. However, because each prior study used variable methods to measure skin, the existing data across the two species could not be reliably compared.

Skin thickness aside, the capacity of skin to detect pain must also depend on the presence and density of the microscopic neural structures of the epidermis. Pain is a complex and primordial physiological function key to survival because it provides a powerful incentive to avoid tissue damage. The basic and absolute need for pain detection is reflected in the remarkable evolutionary conservation of nociception from metazoa to mammals [29]. There is no evidence of significant differences in the neural structures involved in nociception between mammalian species [30]. However, there are gaps in our detailed knowledge concerning presence and density of cutaneous nociceptive fibres in many animal species, including horses.

The capacity of the skin to detect pain normally is recognized to have a relationship with the number or density of nerve fibres in the epidermis, as demonstrated by rare diseases in humans that cause insensitivity to pain [31,32], and the use of intra-epidermal nerve counts to diagnose peripheral sensory neuropathies [23,33]. However, despite the long-standing debate surrounding capacity of horses to experience pain, there are no known reports in the scientific literature that describe and quantify intra-epidermal nerves in the horse.

This study aims to address these knowledge gaps to move us closer to an informed understanding of the capacity of the horse to experience cutaneous pain. This work has been conducted in part to inform the debate surrounding the use of whip strikes in the global horse racing industry, a debate which may become integral to the sustainability of the industry as it moves into the future.

The current project aimed to build on our knowledge of the nociceptive structures of equine skin, particularly in comparison to human skin. The project was designed to test three hypotheses derived from anecdotal approaches to the ability of horses to experience acute superficial pain. First, that horses have thicker epidermal skin layers than humans. Second, that horses have thicker dermal skin layers than humans. Third, that horses have fewer cutaneous nociceptive neurological structures than humans. The current study focused on Thoroughbred horses and horses of Thoroughbred type because of the relevance of the Thoroughbred to racing.

## 2. Materials and Methods

### 2.1. Ethics Approval

This work was approved by Macquarie University Human Anatomy Governance Committee (project number 2019-4). Animal Ethics Committee approval was not required for the use of tissues retrieved post-mortem.

### 2.2. Horse Skin Acquisition

A team of three personnel (one team leader, O.J., and two graduate students) attended an export abattoir to collect the equine tissues for this project. They identified Thoroughbreds and Thoroughbred types (*n* = 20) that were to be processed in a batch. No history was available for the horses. Targeted horses were sexed (mares, *n* = 11; geldings, *n* = 9) and marked with adhesive stickers. After euthanasia, their age was estimated from their dentition and recorded (see Table 1). Skin samples were removed using a scalpel from both the left and right gluteal region and added to container of paraformaldehyde phosphate buffer solution, labelled with the identify code of the horse. They were stored for a minimum of 30 days at room temperature prior to trimming.

### 2.3. Human Skin Acquisition

Human skin was sourced from the Surgical Skills and Anatomy Centre (SSAC), Faculty of Medicine Health and Health Sciences, Macquarie University, NSW, Australia. Samples were acquired from ten fresh frozen human cadavers (men, *n* = 5; women, *n* = 5) and the age of the cadavers recorded (see Table 1). Skin samples were removed from both the left and right gluteal region, and added to a container of paraformaldehyde phosphate buffer solution, labelled with the identify code of the cadaver. They were stored for a minimum of 30 days at room temperature prior to trimming.

While every attempt was made to obtain three samples from each side of each subject, in some cases this was not possible. Missing data were as follows: nerve count data from horse sample #126, both sides (age quartile 1); nerve count, epidermal and dermal data from human sample #44,7 right side (age quartile 3); and nerve count data from human sample #453, both sides (age quartile 3). The problem of missing data was addressed statistically by use of a mixed model with a random effect structure, rather than the solely fixed effect model that had been planned. Mixed models are a method considered appropriate for unbalanced designs and for repeated measures such as was encountered in this data-set.

### 2.4. Histology Processing and Staining

All samples were submitted to a National Association of Testing Authorities (NATA) accredited histology laboratory for sample trimming and slide preparation. Skin from both horses and humans was trimmed into full thickness sections (epidermis to hypodermis inclusive) and placed in cassettes. Samples were embedded in paraffin wax, sectioned at 16 µm and stained with hematoxylin and eosin (HE) using established histological techniques. Slides were then cover-slipped using the adhesive dibutylphthalate polystyrene xylene.

### 2.5. Photographic Images

Photographic images of the horse skin sections stained with hematoxylin and eosin were taken on a BX-51 microscope, using a Jenoptik Gryphax Kapella camera (Jenoptik Group, Jena, Germany). A full thickness (epidermis to hypodermis) image was taken in the centre of each skin section (three on each slide). Images were taken using the panoramic function in the Jenoptik software (Jenoptik Group, Jena, Germany). Each image was 1100 pixels in width, and each pixel represented 1.05887 μm in real distance. Photographic images of the human skin sections stained with hematoxylin and eosin were taken on a CX43 microscope (Olympus, Shinjuku-ku, Tokyo, Japan), using an Olympus EP50 camera (Olympus, Shinjuku-ku, Tokyo, Japan). A full thickness (epidermis to hypodermis) single image or two sequential images were taken in the centre of each skin section (three on each slide), using the EPview software (Olympus, Shinjuku-ku, Tokyo, Japan). Each image was 1100 pixels in width, and each pixel represented 1.18181 μm in real distance. All images were saved as JPEG files, and in total 6 photographs were taken of skin from each horse or human; three from each side of the body. An image of the epidermis and superficial dermis was taken in three sequential regions of the skin section, excluding areas of stain precipitation or tissue folds. Each image was 2080 pixels in width and each pixel was 0.58140 μm in real distance. All images were saved as JPEG files and, in total, six photographs were taken of skin from horse or human; three from each side of the body.

### 2.6. Analysis of H&E Sections

All images were analysed by a specialist veterinary pathologist using Motic Images Plus software (Motic, Kowloon, Hong Kong). Only sections that contained epidermis and dermis through to the hypodermis were analysed. One human sample that did not contain epidermis despite rotation and re-embedding of the tissue and was excluded from analysis. The width of each photographic image was confirmed as 1100 pixels using PixelZoomer software (Matthias Schütz; www.pixelzoomer.com) and each pixel represented 2.9 μm in the photographic software. The image was orientated with the epidermis parallel to the short axis of the image and the area of the epidermis and dermis was measured using the Motic Images Plus freehand tool. In skin samples with invaginations of hair follicles (horse skin), the natural contour of the epidermis was followed across the ostia. In samples with anastomosing rete ridges (human skin), the natural contour of the epidermis was followed. The average depth of the epidermis and dermis was then calculated in microns by dividing the area by the width of the image and corrected for the number of microns per pixel.

### 2.7. Immunohistochemistry Protocol

Paraffin embedded blocks were submitted to a diagnostic histology laboratory for PGP9.5 immunohistochemistry. Three micrometre sections were cut from each block, and three sections were placed on each slide. Immunohistochemical staining PGP9.5 was performed using the Leica Bond III automated staining platform (Leica Biosystems, Melbourne, VIC, Australia). Heat-mediated antigen retrieval (100 °C) was used at pH 9 for 30 min. The primary antibody (Anti-PGP9.5, Polyclonal Rabbit) was incubated for 30 min at ambient temperature. Bond Polymer Refine Red Detection (DS9390) was used with the standard Leica protocol.

### 2.8. Analysis of IHC Sections

The project used standardised and recognized methodology for quantifying intra-epidermal and dermal innervation, as used by the European Federation of Neurological Societies (EFNS) in the assessment of peripheral neuropathies [33]. Morphometric methods were used to quantify the density of neural structures in the epidermis as a count of intra-epidermal nerve fibres per mm of epidermis. The EFNS guidelines [33] were followed to count each nerve fibre crossing the basement membrane at the dermal–epidermal junction as a single unit. Nerve fibres branching in the epidermis were counted as one unit, nerves that split below the basement membrane were counted as two units, nerve fibres that failed to cross the basement membrane or were present in the epidermis but do not cross the basement membrane were not counted. Each fibre to be counted was indicated with a marker, with a total count being recorded for each examined section of skin.

### 2.9. Statistical Analysis

Epidermal depth (µm), dermal depth (µm) and nerve count (epidermal BM/mm), were modelled using a linear mixed effect model, with log transformation of epidermal depth and nerve count because of improved diagnostic plotting following such transformation. The model included the interaction between Species (Horse or Human) and Sex (Female or Male), as well as the age of the subject (the age was split into species quartiles due to the mean and variance differences between species) and the side (Right or Left) from which the sample was taken, both nested within species. Additional to these fixed effects, a fixed section number effect (First, Second and Third) was included to correct for any biases caused by the viewing order of samples and a random individual effect to account for individual variation). Models were fit using R Statistical software [34], making use of the “lme4” package [35]. Statistical significance was accepted at *p* < 0.05.

## 3. Results

### 3.1. Overview

**Figure 1:**
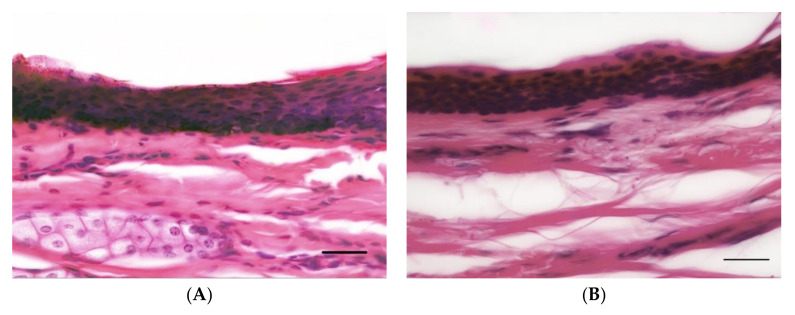
Representative histologic images of horse (**A**) and human (**B**) epidermis and superficial dermis of gluteal skin illustrating comparable epidermal thickness (Haematoxylin and Eosin, 400×, 16 μm sections. Bar = 20 μm).

Histological examination of horse and human skin revealed similar anatomical architecture with clear demarcation between the epidermis, dermis and hypodermis and shared fundamental histological features. The epidermis of stratified squamous epithelium contained the stratum basale embedded with variable numbers of pigmented cells on a basement membrane, stratum granulosum, stratum spinosum and stratum corneum. The superficial papillary dermis extended from the epidermal basement membrane and consisted of loosely separated collagen bundles that surrounded primary hair follicles and associated adnexal glandular tissue. The deeper reticular dermis extended from the follicular–adnexal units and consisted of more tightly packed collagen bundles. The hypodermis contained a predominance of adipose tissue with loose strands of collagen. Observation differences between the horse and human skin included greater density of follicular-adnexal units in the horse. While both equine and human epidermis form distinct rete ridges at the dermo-epidermal junction, these tended to be thinner and deeper with occasional anastomosis noted in human skin (see Figure 1).

### 3.2. Epidermal Thickness

**Figure 2:**
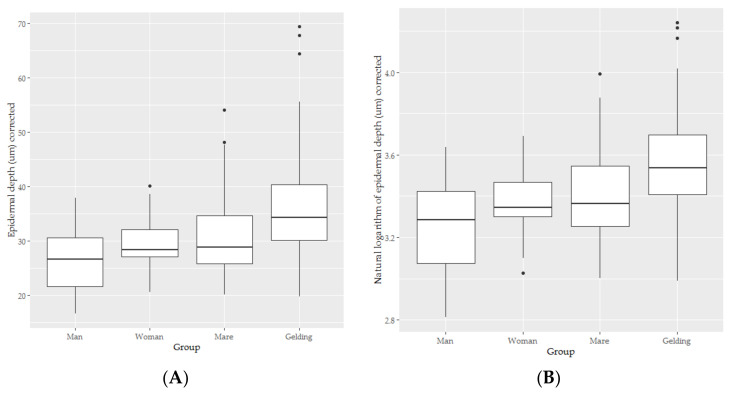
The box-and-whisker plots for the epidermal depth (**A**) and natural logarithm of epidermal depth (**B**) of skin from men (*n* = 5), women (*n* = 5), mares (*n* = 11) and geldings (*n* = 9).

Plots of the data showed some signs of a right skew, so introduction of a logarithmic transformation was decided upon. The logarithmic boxplots are presented for reference purposes (see Figure 2).

The marginal R^2^ and conditional R^2^ for the epidermal thickness model were 0.216 and 0.585, respectively, indicating that both fixed effects (marginal R^2^) and fixed and random effects considered together (conditional R^2^) account for substantial proportions of the variance in epidermal depth. The individual random effect for the epidermis model accounted for 47% of the variance. The fixed effect coefficients calculated for this model appear in Table 2.

**Figure 3:**
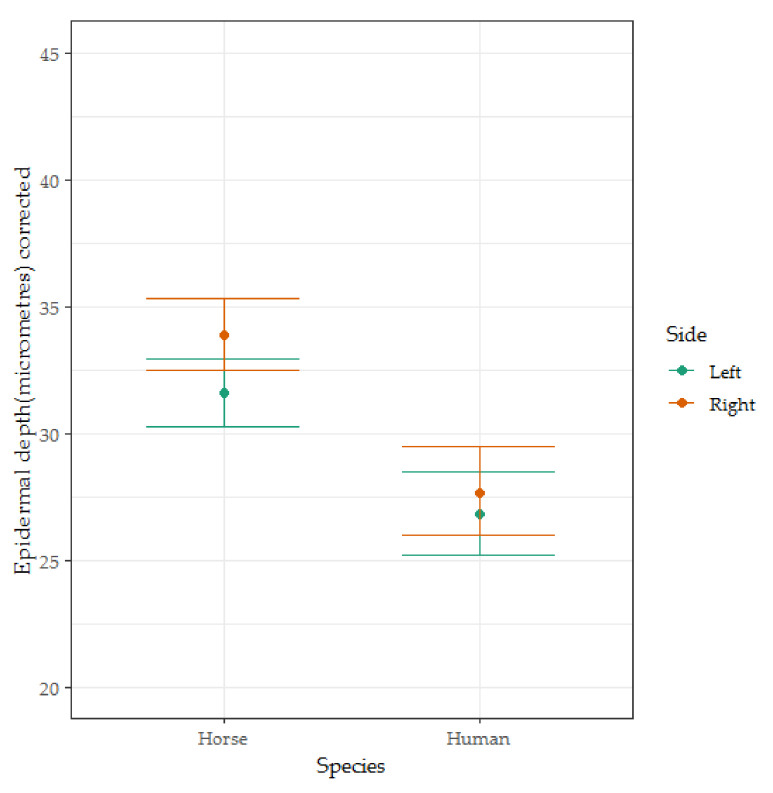
The box-and-whisker plots for the corrected epidermal depth of skin from men (*n* = 5), women (*n* = 5), mares (*n* = 11) and geldings (*n* = 9). Coefficients and error bars are shown on the original measurement scale for ease of interpretation. This figure illustrates the significant interaction effect found between the side from which the sample was taken and the species from which the sample was taken.

No significant differences were found between humans and horses for left sides samples (*t* = 0.117, *p* = 0.908, see Figure 3). Humans had skin 0.185 units thinner on the log scale which corresponds to approximately 16.9% thinner.

### 3.3. Dermal Thickness

**Figure 4:**
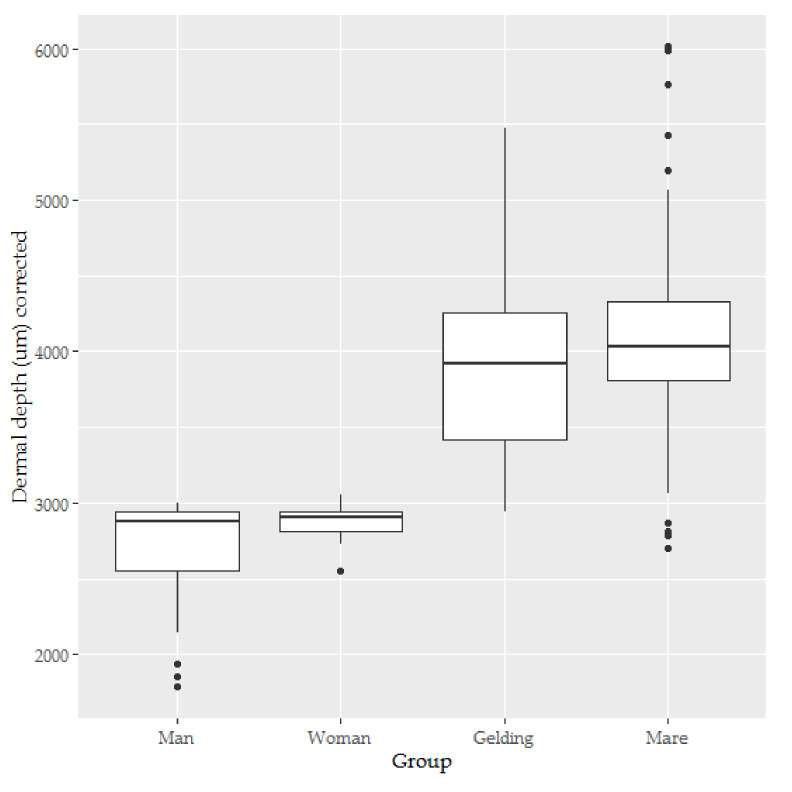
The box-and-whisker plots for the dermal depth of skin from men (*n* = 5), women (*n* = 5), mares (*n* = 11) and geldings (*n* = 9).

There was some suggestion of negative skew (see Figure 4), but the diagnostic residual plots were adequate.

The marginal R2 and conditional R2 for the dermal thickness model were 0.548 and 0.867, respectively, indicating that both fixed effects (marginal R2) and fixed and random effects considered together (conditional R2) account for substantial proportions of the variance in dermal depth. The individual random effect for the dermis model accounted for 71% of the variance. The fixed effect coefficients calculated for this model appear in Table 3.

**Figure 5:**
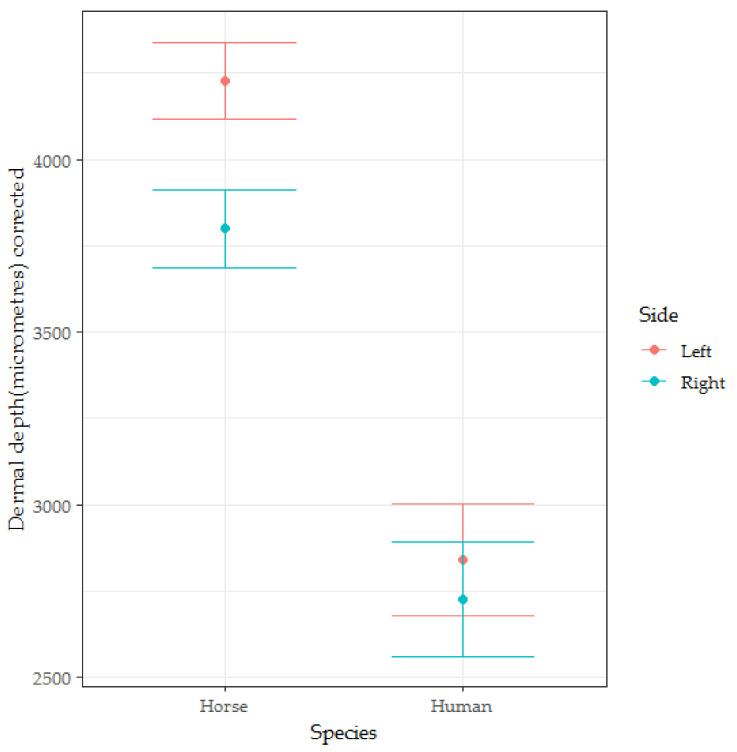
The box-and-whisker plots for the dermal depth of skin from men (*n* = 5), women (*n* = 5), mares (*n* = 11) and geldings (*n* = 9). Coefficients and error bars are shown on the original measurement scale for ease of interpretation. This figure illustrates the significant interaction effect found between the side from which the sample was taken and the species from which the sample was taken.

The human dermis was significantly thinner than the horse dermis (*t* = −2.946, *p* = 0.007, see Figure 5).

### 3.4. Epidermal Nerve Count

**Figure 6:**
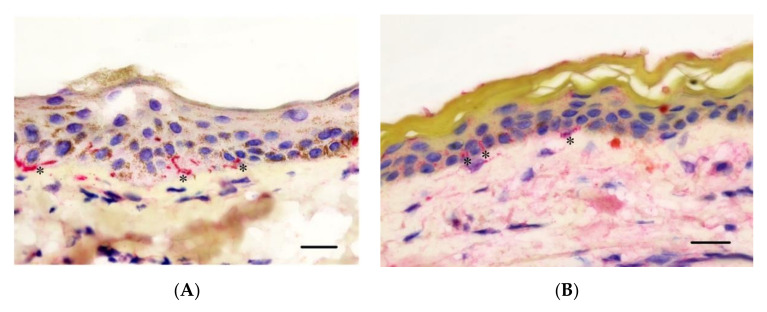
Representative skin sections stained with immunohistochemistry using anti-PGP9.5 antibodies from Horse (**A**) and Human (**B**) skin. Images include epidermis and superficial dermis. Nerve endings are selectively stained with a red dye (*). Sections illustrate the finding that epidermal nerve endings are found in equivalent density across horse and human skin (anti-PGP9.5 immunohistochemistry, 400× magnification. Bar = 20 μm).

**Figure 7:**
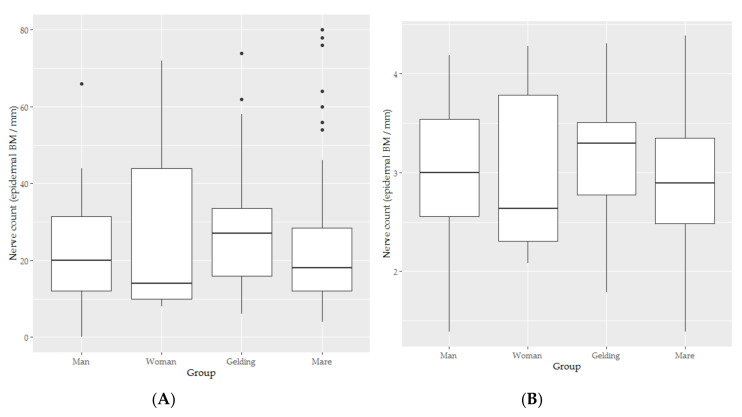
The box-and-whisker plots for the epidermal nerve count (**A**) and natural logarithm of epidermal depth (**B**) of skin from men (*n* = 5), women (*n* = 5), mares (*n* = 11) and geldings (*n* = 9).

Typical examples of the results of the immunohistochemistry studies are shown in Figure 6. The distributions of nerve counts scores appear in Figure 7.

Some concerning shapes on the QQ and Scale location plots were found for in the residuals of the linear mixed model of the untransformed data, but were ameliorated with log transformation, apparently due to the presence of true zeros and positive skew in the original data. 

The marginal R2 and conditional R2 for the model (see Table 4) of the transformed nerve count data (see Figure 7, panel B) were 0.042 and 0.716 respectively, indicating that both fixed effects (marginal R2) were relatively unimportant for this model but random individual effect still accounted for substantial proportions of the variance in epidermal nerve count, in this case, 70% of the variance. The relative unimportance of the fixed effects, which include Species compared to the thickness of skin layers is interesting to note. The coefficients calculated for this model appear in Table 4.

**Figure 8:**
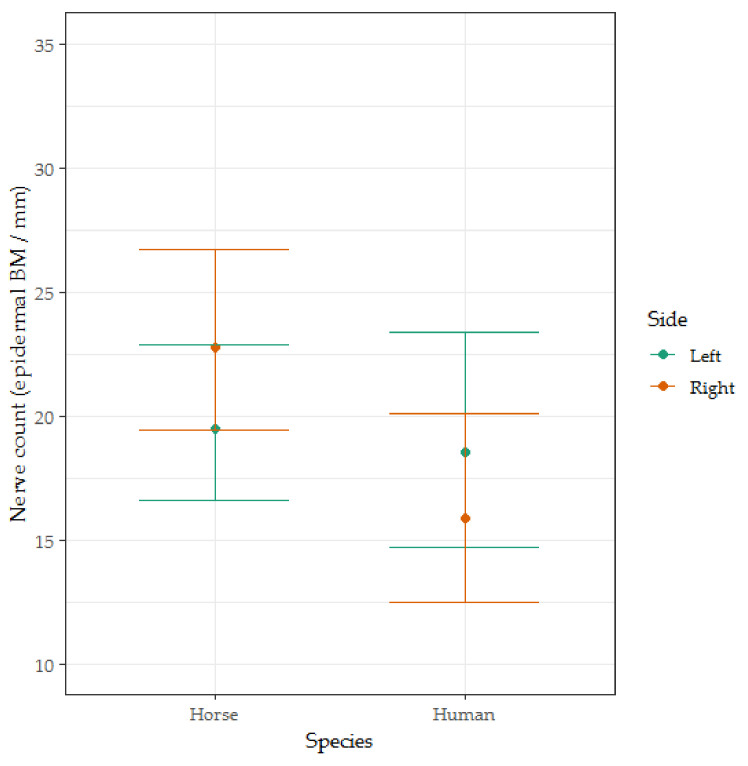
The box-and-whisker plots for the epidermal nerve count of men (*n* = 5), women (*n* = 5), mares (*n* = 11) and geldings (*n* = 9). Coefficients and error bars are shown on the original measurement scale for ease of interpretation. This figure illustrates the significant interaction effect found between the side from which the sample was taken and the species from which the sample was taken.

There was no significant difference between human and horse nerve counts (*t* = 0.051, *p* = 0.960, see Figure 8).

## 4. Discussion

The results revealed no significant differences between epidermal thickness of humans and horses for left-sided (reference) samples. This refutes our first hypothesis, that horses have thicker epidermal skin layers than humans. There was mild asymmetry of the thickness of the horse epidermis between left and right sides, and the right horse epidermis was therefore marginally thicker than right human epidermis. This is an unusual finding and is discussed below. As was expected, the horse dermis was significantly thicker than the human dermis, supporting our second hypothesis. The structural integrity of the relatively thick equine dermis may withstand forceful impacts better than human dermis. However, the facts of cutaneous innervation with respect to nociception demonstrate that dermal thickness cannot be a significant factor in skin sensitivity. There were no significant differences between the epidermal nerve counts of humans and horses, refuting the third hypothesis that horses have fewer dermal nociceptive neurological structures than humans.

Together, these findings indicate that horse skin is virtually indistinguishable from human skin with respect to the basic anatomical structures relevant to cutaneous pain detection. These observations must refresh old assumptions made about the capacity of the “thick-skinned” horse to experience pain in comparison to humans.

It is recognized that pain is an enormously complex process, with huge amount of modulation of nociceptive signalling between noxious stimulation at the skin and the perception of pain in the higher centres of the central nervous. The current study does not attempt to assume, based on these findings, that the end-experience of pain in the horse is the same as in humans, as such a comparison is currently outside the capability of science, even when comparing the experience of pain between two humans. However, this does mean that broad similarity between species permits valid comparisons about pain perception, even in the absence of absolute equivalence of skin structures.

An observational study of horses racing in Australia [4] found 83% of whip strikes caused indentations of the skin of the horses whipped, and comparative studies in mice and humans showed such deformation is likely to be detected by cutaneous nociceptors [36], as did a recent study in horses [37]. Fundamentally, if an anti-nociceptive/analgesic state arises in all racing horses, it begs the question that if the whip has lost its salience, then why use it at all? Recent evidence indicates that certain distinct stress-related, affective states are characterised by enhanced, not reduced pain perception [38,39,40,41].

Repeated whipping is common in racing even though the IFHA forbids “using the whip with excessive frequency” [42]. The accumulated damage from successive strikes is likely to create more pain and inflammation. This assumption is based on the results of a feline study [43] in that repeated application reduced the thresholds for nociceptor activation and enhanced the duration of their responses. In Australia, racing thoroughbreds may be struck no more than five times prior to the final 100 m but after this point they can be struck at the jockey’s discretion [44]. This can equate to a strike per stride and effectively this means that 18 strikes per race are within the rules [13].

Forceful impact to equine skin may occur outside racing and other forms of equestrian activity. It is accepted that horses can cause severe injuries to one another, for example in disputes over focal resources such as food [14]. However, an important distinction to make in this context is that they are not being exposed to harm or injury by commission of an act by a human that is of no benefit to the horse.

In the current study, the horse epidermis was deeper on the right than on the left- and right-sided horse epidermis was thicker than human epidermis (which showed a non-significant trend towards similar left–right asymmetry). Bilateral asymmetry of epidermal thickness is a highly unexpected finding. The authors are not aware of reported asymmetry for measurements of normal skin thickness (epidermis or dermis) in any human or mammalian study. Localised or regional thickening of the epidermis may occur in pathologic states, and is known as epidermal hyperplasia or acanthosis. Epidermal thickening occurs in response to chronic insult or injury to the epidermis [45]. Thinning of the epidermis can occur in pathological states. However, these are generally restricted to cutaneous endocrinopathies and the epidermal thinning in these conditions is bilaterally symmetrical. Other pathological or physiological causes cannot be entirely excluded. There is evidence that horses tend to use their left eye when approaching potential threats [46], which could arguably eventuate in a bias for left-sided injury. So, one might have predicted that the skin on the left side of horses needs to be stronger than that on the right side. An investigation into epidermal asymmetry in horse skin would be a worthwhile future study.

The role of pain in horse sports is part of the growing debate about what constitutes ethical equitation [7,47]. In a flight animal, such as the horse, being unable to resolve aversive cutaneous stimulation causes distress. Horses have evolved to run away from such stimuli since the most likely natural cause of such stimulation is contact from a predator. Repeated strikes of the whip in horses that are fatigued as they end a race [3] are likely to be distressing and cause suffering. The horses’ loss of agency is described in the literature as helplessness and repeated, inescapable, treatment of this sort is thought to lead to learned helplessness [48].

The concept of ethical equitation [1] advocates a three-step process for stakeholders who seek to retain the social license to operate [13]. It demands that we identify the causes of distress in the horses we ride, that we mitigate these stressors as much as possible and we justify the retention of those that cannot be mitigated. The current findings suggest the ethical future for racing is one in which whips are carried but not used for so-called encouragement. Fortunately, this model is already well established in the UK where apprentice jockeys compete in hands-and-heels series [49].

A minor limitation of the current study is that human cadavers were fresh frozen, while the equine tissues were processed immediately after euthanasia. It is possible, but highly unlikely, that this contributed to the similarity of the tissues. The imbalance between the numbers of human and equine samples was caused by the 2020 national lockdown in Australia that interrupted access to specimens from human anatomy classes. A further minor limitation is that we were unable to gather data on any potential structural difference in the hairs of horses and humans. It appears unlikely that hairs afford meaningful protection from the impacts of whips, given that whips cause cavitation waves through the skin and underlying tissues [50]. Studies of anaesthetized horses may assist in this domain and might also be helpful in revealing the thermographic responses of horse skin to whipping. Future adjunctive research should include histopathological examination of bilateral skin and subcutaneous tissues from horses which have died within seven days of a racing event. This could reveal any evidence of inflammation and repair at the site of recent whip strikes.

## 5. Conclusions

This primary aim of this project was to improve our understanding of the neuroanatomy of horse skin with respect to cutaneous pain sensation. The project achieved this aim through quantitative methods, using research material that can be openly accessed by interested parties. The results revealed no significant difference between the epidermal nerve counts of humans and horses. There were no significant differences between epidermal thickness of humans and horses for left side samples. The human dermis was significantly thinner than the horse dermis. The horse epidermis was deeper on the right than the left. These findings indicate that the superficial pain-sensitive epidermal layer of horse skin is as richly innervated and is of equivalent thickness as human skin, demonstrating that humans and horses have the equivalent key anatomical structures to detect cutaneous pain.

## Figures and Tables

**Table 1:** The distribution age ranges of men (*n* = 5), women (*n* = 5), mares (*n* = 11), and geldings (*n* = 9), from which six sections were taken.

Quartile	Horse Age Range	Number of Horse Sections	Human Age Range	Number of Human Sections
1	3–5	24	52–61	12
2	8–10	42	67–77	18
3	11–15	30	83–87	18
4	16–25	30	92–94	12

**Table 2:** The fixed effect coefficients calculated for a linear mixed model exploring the epidermis of men (*n* = 5), women (*n* = 5), mares (*n* = 11) and geldings (*n* = 9). Significant findings appear in bold.

	Coefficient	Std. Error	d.f.	*t* Value	*p* Value
(Intercept)	3.499	0.127	28.898	27.478	<2 × 10^-16^
SpeciesHuman	−0.185	0.218	25.565	−0.849	0.404
SexMale	0.132	0.083	24.748	1.577	0.128
Section	0.005	0.016	148.977	0.346	0.730
SpeciesHuman:SexMale	−0.228	0.145	25.346	−1.572	0.128
SpeciesHorse:Age_quantile	−0.049	0.039	24.748	−1.238	0.227
SpeciesHuman:Age_quantile	0.004	0.056	24.790	0.076	0.940
SpeciesHorse:SideRight	**0.071**	**0.031**	**148.977**	**2.291**	**0.023**
SpeciesHuman:SideRight	0.032	0.047	152.012	0.694	0.489

**Table 3:** The coefficients calculated for a linear mixed model exploring the dermis of men (*n* = 5), women (*n* = 5), mares (*n* = 11) and geldings (*n* = 9). Significant findings appear in bold.

	Estimate	Std. Error	d.f.	*t* Value	*p* Value
(Intercept)	4358.564	343.092	26.694	12.704	0.000
SpeciesHuman	**−1759.220**	**597.193**	**25.335**	**−2.946**	**0.007**
SexMale	−193.894	229.847	24.993	−0.844	0.407
Section	−10.844	27.747	149.095	−0.391	0.696
SpeciesHuman:SexMale	91.974	397.560	25.280	0.231	0.819
SpeciesHorse:Age_quantile	−5.897	108.129	24.993	−0.055	0.957
SpeciesHuman:Age_quantile	124.633	154.417	25.013	0.807	0.427
SpeciesHorse:SideRight	**−428.025**	**54.606**	**149.095**	**−7.838**	**0.000**
SpeciesHuman:SideRight	−113.599	83.199	150.535	−1.365	0.174

**Table 4:** The coefficients calculated for a linear mixed model exploring the epidermal nerve count of men (*n* = 5), women (*n* = 5), mares (*n* = 11) and geldings (*n* = 9). Significant findings appear in bold.

	Estimate	Std. Error	d.f.	*t* Value	*p* Value
(Intercept)	2.727	0.468	24.627	5.829	0.000
SpeciesHuman	0.041	0.806	24.215	0.051	0.960
SexMale	0.238	0.312	23.887	0.764	0.453
Section2	0.081	0.076	142.990	1.072	0.286
Section3	0.080	0.076	142.990	1.057	0.292
SpeciesHuman:SexMale	−0.254	0.534	24.160	−0.476	0.638
SpeciesHorse:Age_quantile	0.049	0.150	23.887	0.329	0.745
SpeciesHuman:Age_quantile	0.063	0.206	23.907	0.303	0.765
SpeciesHorse:SideRight	**0.150**	**0.075**	**142.990**	**1.993**	**0.048**
SpeciesHuman:SideRight	−0.148	0.112	144.384	−1.331	0.185
